# Time series analysis of fine particulate matter and asthma reliever dispensations in populations affected by forest fires

**DOI:** 10.1186/1476-069X-12-11

**Published:** 2013-01-28

**Authors:** Catherine T Elliott, Sarah B Henderson, Victoria Wan

**Affiliations:** 1 British Columbia Center for Disease Control, Environmental Health Services, BC Centre for Disease Control, Main Floor, 655 12th Ave W, Vancouver, BC, V5Z 4R4, Canada; 2 University of British Columbia School of Population and Public Health, 2206 East Mall, Vancouver, BC, V6T 1Z3, Canada

**Keywords:** Fires, Smoke, Air pollution, Asthma, Pulmonary disease chronic obstructive, Epidemiology

## Abstract

**Background:**

Several studies have evaluated the association between forest fire smoke and acute exacerbations of respiratory diseases, but few have examined effects on pharmaceutical dispensations. We examine the associations between daily fine particulate matter (PM_2.5_) and pharmaceutical dispensations for salbutamol in forest fire-affected and non-fire-affected populations in British Columbia (BC), Canada.

**Methods:**

We estimated PM_2.5_ exposure for populations in administrative health areas using measurements from central monitors. Remote sensing data on fires were used to classify the populations as fire-affected or non-fire-affected, and to identify extreme fire days. Daily counts of salbutamol dispensations between 2003 and 2010 were extracted from the BC PharmaNet database. We estimated rate ratios (RR) and 95% confidence intervals (CIs) for each population during all fire seasons and on extreme fire days, adjusted for temperature, humidity, and temporal trends. Overall effects for fire-affected and non-fire-affected populations were estimated via meta-regression.

**Results:**

Fire season PM_2.5_ was positively associated with salbutamol dispensations in all fire-affected populations, with a meta-regression RR (95% CI) of 1.06 (1.04-1.07) for a 10 ug/m^3^ increase. Fire season PM_2.5_ was not significantly associated with salbutamol dispensations in non-fire-affected populations, with a meta-regression RR of 1.00 (0.98-1.01). On extreme fire days PM_2.5_ was positively associated with salbutamol dispensations in both population types, with a global meta-regression RR of 1.07 (1.04 - 1.09).

**Conclusions:**

Salbutamol dispensations were clearly associated with fire-related PM_2.5_. Significant associations were observed in smaller populations (range: 8,000 to 170,000 persons, median: 26,000) than those reported previously, suggesting that salbutamol dispensations may be a valuable outcome for public health surveillance during fire events.

## Background

The public health effects of acute environmental exposures are often described as a pyramid, with the rarest outcomes at the peak and the more common outcomes at the base. The rarest outcomes are most severe, while the most common outcomes are the mildest. Many population-based studies focus on the upper part of the pyramid because severe outcomes are typically recorded in administrative databases. However, their rarity makes it challenging to evaluate short-lived exposures with adequate statistical power, even in large populations. In the case of forest fire smoke, only two [1,2] of five [3-5] studies have reported significant associations between smoke-related particulate matter (PM) and all-cause mortality, and effects specific to respiratory mortality were not clear. Similarly, time-series studies have reported significant associations between smoke-related PM and respiratory hospital admissions in multiple settings [6-10], but most have been conducted in large towns or cities, and not in the remote and rural areas most affected by fire smoke.

The effects of forest fire smoke on milder health outcomes have generally been examined in smaller panel studies [11-14] due to the absence of population-based information. However, administrative databases that capture common health outcomes could serve to advance forest fire smoke epidemiology by allowing us to study smaller populations and to detect smaller effect estimates with the increased statistical power. Electronic registries of pharmaceutical dispensations provide such data, and have previously been used to evaluate the public health impacts of other short-lived events, such as human [15] and natural [16] disasters. Short-acting beta-agonist (SABA) dispensations are specifically associated with acute exacerbations of obstructive lung diseases (such as asthma and chronic obstructive pulmonary disease), and they outnumber severe outcomes [17]. As such, SABA dispensations may be a more sensitive indicator of obstructive lung disease exacerbations within the population [18].

The Canadian province of British Columbia (BC) is regularly impacted by forest fires. It has both a comprehensive pharmaceutical database and a long-standing air quality monitoring network that covers many smaller communities. This setting provides a valuable opportunity to study the public health effects of forest fire smoke in smaller populations using a mild health outcome. Here we examine the associations between daily PM_2.5_ (PM less than 2.5 microns in diameter) and dispensations of medications used to relieve exacerbations of chronic respiratory diseases in fire-affected versus non-fire-affected populations between 2003 and 2010.

## Methods

### Study area

**Figure 1:**
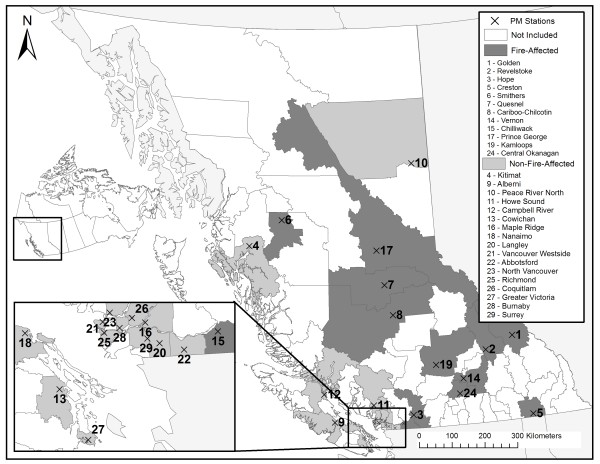
**Map of the study region showing the local health areas (LHAs) included in the study, and the locations of the PM air quality monitoring stations.** Study LHAs are numbered in order of increasing population, as in Table 1.

The study was conducted in the province of British Columbia (BC), on the west coast of Canada. Forest fires burn an average of 980 km^2^ per year in BC [19], and widespread infestation by the mountain pine beetle has left forests more susceptible to extreme events in recent years [20]. The province is geographically divided into 89 local health areas (LHAs), ranging in size from 40 – 130,000 km^2^ (Figure 1), and in 2006 population from 542 – 352,783 people [21]. Geographically smaller LHAs typically have larger populations living in urban and suburban areas, while larger LHAs have smaller populations living in rural and remote communities.

### Exposure assessment

The air quality monitoring network in BC is maintained by the BC Ministry of Environment. Ambient concentrations of particulate matter (PM) are continuously measured at several stations by PM_2.5_ and/or PM_10_ (PM less than 10 microns in diameter) tapered element oscillating microbalances [22]. These instruments are heated to 30 or 40°C depending on their locations, and loss of volatile materials is expected to be less during the fires season than during the winter months [23]. We started by identifying all stations that had PM_2.5_ and/or PM_10_ measurements for every fire season (April 1 through September 30) in the study period. Any LHA with one or more of these stations was included in the study (Figure 1). For LHAs with multiple stations, the one closest to the LHA population center was used. As such, the daily PM exposure of the population within each study LHA was estimated using data from a single monitoring station within that LHA.

All PM data were converted to PM_2.5_ concentrations. For LHAs where PM_2.5_ measurements were available for the whole period, those data were used. For LHAs with some PM_2.5_ and some PM_10_ measurements, we adjusted PM_10_ to PM_2.5_ using the regression coefficients from linear models applied to all fire seasons when both instruments were running simultaneously. For LHAs with PM_10_ measurements only, or with insufficient overlap between instruments, we used the time-weighted average of linear regression coefficients from the other stations with simultaneous PM_2.5_ and PM_10_ measurements.

### Fire-affected LHAs and extreme fire days

The air quality monitoring network measures PM contributions from all sources, including smoke from forest fires. We focused on the effects of fire smoke by using data from the Moderate Resolution Imaging Spectroradiometers (MODIS) to classify LHAs as fire-affected and non-fire-affected, and to identify extreme fire days,. These remote sensing instruments overpass most areas of the Earth four times daily, detecting fires at a resolution of 1 km^2^ at nadir [24]. The information recorded for each detected fire includes its central latitude and longitude, and a measure of its intensity, known as the fire radiative power (FRP, in MW). The FRP is proportional to aerosol emissions, and serves as a good indicator of the smoke generated by the detected fire [25-27]. We downloaded these data for BC and its surrounding areas from the Fire Information Resource Management System (FIRMS) [28], and used a geographic information system (GIS) to map all of the fires detected during the study period.

**Figure 2:**
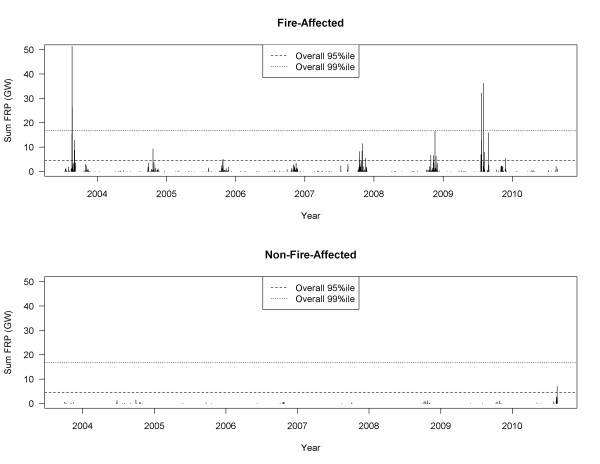
Examples of the time series of summed fire radiative power (FRP, in gigawatts) values for a fire-affected LHA (above, Central Okanagan) and a non-fire-affected LHA (below, Kitimat).

To assess the impact of fire on each LHA we used the GIS to draw a 100 km radius circle around its PM monitoring station, and then calculated the daily sum of FRP from all fires detected within that circle. Next, we aggregated daily FRP sums for all LHAs to examine the percentiles of the overall distribution. Finally, an LHA was defined as fire-affected if the plotted time-series of daily FRP values showed that the overall 95th percentile was exceeded in three or more of the nine fire seasons (Figure 2). To identify extreme fire days we summed all FRP values detected within and around BC for each day of the study period, and used the 80th, 90th, and 95th percentiles of the distribution to limit analyses to periods most likely affected by heavy smoke.

### Pharmaceutical dispensations

Daily counts of pharmaceutical dispensations were received for each LHA from the BC PharmaNet database. Law requires that every prescription dispensed in the province be recorded in PharmaNet, regardless of the recipient or the payer [29]. We decided *a priori* to examine relationships between PM_2.5_ and counts for inhaled salbutamol sulfate, a selective beta-2-adrenoreceptor agonist that is commonly and specifically used to rapidly relieve exacerbations of asthma, COPD, and other obstructive lung diseases. Dispensations included all inhaled preparations of salbutamol available in BC (i.e. aerosol inhalers, powder inhalers and nebulizer solutions). Salbutamol preparations for ingestion were excluded. Other selective beta-2-adrenergic inhalants were also excluded, because preliminary analyses showed that they were rarely prescribed in BC (less than 5% of dispensations).

### Time-series models

For every LHA included in the study we estimated the effect of daily PM_2.5_ concentrations on the rate of pharmaceutical dispensations for respiratory reliever medications, during all fire seasons and on extreme fire days. We used generalized linear models with natural cubic splines, adjusted for temperature, relative humidity, and temporal trends as shown in Equation 1.

$$O_{t}˜\mathrm{Poisson}\left(\mu_{t},\sigma^{2}\right)$$

$$g\left(\mu_{t}\right)=PM_{\mathrm{lag}01}+\mathrm{ns}\left(T_{t},\mathrm{df}=3\right)+\mathrm{ns}\left(RH_{t},\mathrm{df}=3\right)+\mathrm{YMDO}W_{t}$$

Where: *O*_*t*_ = observed dispensation count in the LHA on day *t*; *PM*_*lag01*_ = PM_2.5_ concentration in the LHA averaged over days *t* and *t-1*.; *T*_*t*_ = mean temperature in the region of the LHA on day *t*, fitted as a natural cubic spline with three degrees of freedom; *RH*_*t*_ = mean relative humidity in the region of the LHA on day *t*, fitted as a natural cubic spline with three degrees of freedom; *YMDOW*_*t*_ = year, month, and day of week (statutory holidays treated as Sundays) on day *t*, fitted as a factor variable with 378 levels (9 years * 6 months * 7 days). The resulting estimate of effect was the rate ratio (RR) associated with a 10 ug/m^3^ increase in PM_2.5_. A lag of 0-1 days was chosen for principal analyses based on model fit statistics from preliminary analyses, but lags of 0 through 7 days were tested. All analyses were completed in the R statistical computing environment [30]. After fitting individual models for each LHA, we conducted a random effects meta-regression using the inverse variance method [31] to estimate an overall effect for the fire-affected LHAs and the non-fire-affected LHAs during the fire season and on extreme fire days.

## Results

### Included LHAs

A total of 29 (out of 89) LHAs were included in the study (Table 1 and Figure 1). Their land areas ranged from 48 to 76,215 km^2^ and their 2006 populations ranged from 7,024 to 352,783 people. The average daily salbutamol dispensations ranged from 4.3 to 103.4 (Table 1). Most of the LHAs had PM_2.5_ measurements covering the majority of the study period, and PM_10_ concentrations were converted to PM_2.5_ with coefficients ranging from 0.27 to 0.69, with an average of 0.49. Mean fire season PM_2.5_ concentrations ranged from 2.8 to 11.8 ug/m^3^ across all stations. Maximum concentrations ranged from 33.4 to 248.1 ug/m^3^ for the 12 LHAs classified as fire-affected, and from 15.2 to 49.3 ug/m^3^ for the 17 LHAs that were not-fire-affected (Table 1). The mean PM_2.5_ concentrations on fire days in the 80th, 90th, and 95th percentiles of provincial FRP were 8.2, 9.6, and 11.2 ug/m^3^, respectively. In the 95th percentile there were 28 extreme fire days in 2003, 22 in 2009, 21 in 2010, 18 in 2004, 7 in 2006, 2 in 2007, and none in 2005 or 2008.

**Table 1:** Summary information for local health areas (LHAs) included in the analyses, listed in order of 2006 population

**LHA name**	**2006**^**a**^**population**	**Daily average salbutamol dispensations (fire season)**	**Area (km**^**2**^**)**	**Fire season PM**_**2.5**_	**Fire season PM**_**10**_	**PM**_**2.5**_**/PM**_**10**_**coefficient**	**Fire season Mean PM**_**2.5**_**(μg/m**^**3**^**)**	**Fire season Max PM**_**2.5**_**(μg/m**^**3**^**)**	**Fire- affected?**
Golden	7,024	4.5	13,350	2003-2010	2003-2010	0.51	5.7	74.0	Y
Revelstoke	7,897	4.3	9,307	2007-2010	2003-2007	0.49^b^	7.1	81.7	Y
Hope	8,062	4.9	5,280	2004-2010	2003-2010	0.48	5.0	33.4	Y
Kitimat	10,443	5.1	19,639	2003-2010	2003-2010	0.44	2.8	24.8	
Creston	11,917	6.8	3,789	2010	2003-2009	0.49 ^b^	7.3	48.9	Y
Smithers	16,073	7.1	9,827	2005-2010	2003-2010	0.27	4.2	66.8	Y
Quesnel	22,930	11.7	23,732	2003-2010	2003-2010	0.69	7.4	139.4	Y
Cariboo-Chilcotin	26,150	12.9	44,695	2003-2010	2003-2010	0.59	6.1	248.1	Y
Alberni	31,077	14.0	6,809	-	2003-2010	0.49 ^b^	4.8	15.2	
Peace River North	32,642	14.4	68,765	-	2003-2010	0.49 ^b^	11.8	49.3	
Howe Sound	32,327	12.6	9,236	-	2003-2010	0.49 ^b^	8.3	33.7	
Campbell River	40,173	18.0	13,624	2006-2010	2003-2009	0.50	3.9	41.7	
Cowichan	54,855	22.9	735	2010	2003-2009	0.49 ^b^	4.8	37.9	
Vernon	62,227	29.9	5,555	2003-2010	2003-2008	0.38	5.4	130.8	Y
Chilliwack	79,302	36.2	1,314	2003-2010	2003-2010	0.40	5.3	35.7	Y
Maple Ridge	88,020	32.3	1,450	2003-2010	2003-2010	0.45	5.5	36.5	
Prince George	94,852	44.3	76,215	2003-2010	2003-2010	0.53	7.4	176.4	Y
Nanaimo	98,561	46.5	1,289	2003-2010	-	-	3.7	48.7	
Kamloops	105,491	44.8	16,319	2003-2010	2003-2008	0.49	5.5	140.1	Y
Langley	122,219	46.7	323	2003-2010	2003-2010	0.51	5.5	34.0	
Vancouver Westside	129,011	24.6	48	2004-2010	2003-2008	0.52	5.5	32.1	
Abbotsford	130,008	51.1	413	2010	2003-2010	0.49 ^b^	6.9	23.9	
North Vancouver	134,453	33.3	398	-	2003-2010	0.49 ^b^	6.4	27.2	
Central Okanagan	167,323	59.1	2,942	2003-2010	2003-2008	0.56	5.5	185.6	Y
Richmond	182,652	37.5	124	2003-2010	2003-2010	0.51	5.0	40.3	
Coquitlam	205,495	52.6	733	2004-2010	2003-2008	0.50	6.2	42.0	
Burnaby	210,507	56.4	90	2003-2010	2003-2010	0.45	5.2	42.7	
Greater Victoria	217,374	65.4	113	2003-2010	-	-	5.0	17.3	
Surrey	352,783	103.4	333	-	2003-2010	0.49 ^b^	7.1	19.1	

The forest fire season is defined as April 1 – September 30 each year.

^a^ 2006 was a national census year, and was also the midpoint of the study period.

^b^ Average from all other stations used.

### Associations between PM_2.5_ and salbutamol dispensations

**Figure 3:**
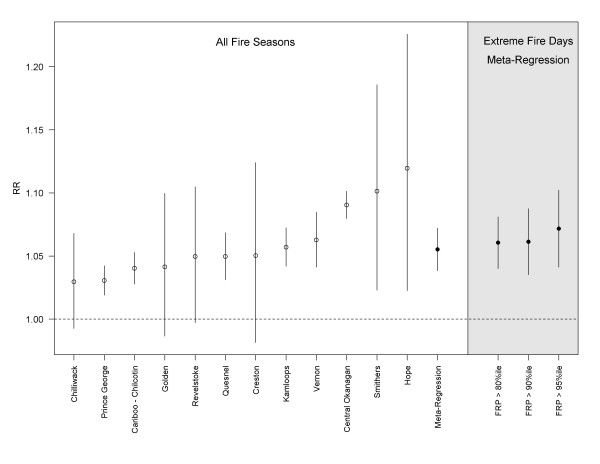
**Regression results for the association between a 10 ug/m**^**3**^**increase in PM**_**2.5**_**(day-of and day-before average, lag**_**01**_**) and dispensation counts for the respiratory relief medication salbutamol sulfate in fire-affected local health areas (LHAs).** Results for individual LHAs are ordered by the rate ratio (RR) point estimates for all fire seasons, followed by the meta-regression estimates for all fire seasons, and extreme fire days in the 80th, 90th, and 95th percentiles.

**Figure 4:**
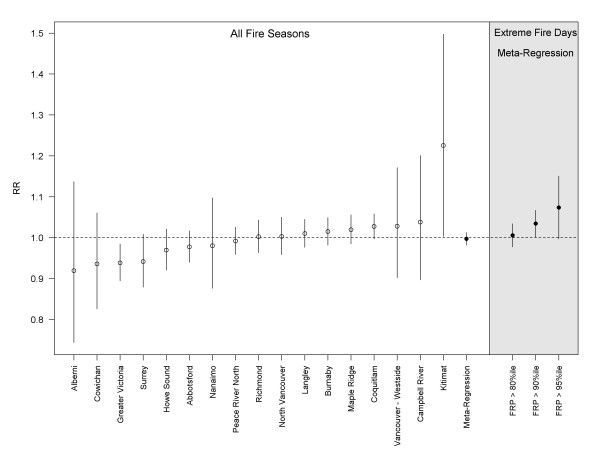
**Regression results for the association between a 10 ug/m**^**3**^**increase in PM**_**2.5**_**(day-of and day-before average, lag**_**01**_**) and dispensation counts for the respiratory relief medication salbutamol sulfate in non-fire-affected local health areas (LHAs).** Results for individual LHAs are ordered by the rate ratio (RR) point estimates for all fire seasons, followed by the meta-regression estimates for all fire seasons, and extreme fire days in the 80th, 90th, and 95th percentiles.

Fire season PM_2.5_ was positively associated with salbutamol dispensations in all fire-affected LHAs, with statistically significant results in 8 of 12 cases (Figure 3). The meta-regression RR (95% confidence interval) for a 10 ug/m^3^ increase in PM_2.5_ was 1.06 (1.04 – 1.07). The effect was evident at lags up to four days, decreasing to null by the fifth day. Fire season PM_2.5_ was not significantly associated with salbutamol dispensations in 15 of 17 non-fire-affected LHAs (Figure 4), with a meta-regression RR (95%CI) of 1.00 (0.98 – 1.01). The two exceptions were a protective effect observed in Greater Victoria, and a positive association of 1.23 (1.00 – 1.49) in Kitimat, where an aluminum smelter is an important source of PM and other air emissions. When analyses were restricted to fire days in the 80th, 90th, and 95th percentile of the provincial sum of FRP, the meta-regression RRs remained stable for the fire-affected LHAs (Figure 3), but increased with each restriction for the non-fire-affected LHAs (Figure 4). The point estimate was the same for both population types at the 95th percentile, with wider confidence intervals for the non-fire-affected group. The province-wide meta-regression estimate was 1.07 (1.04 – 1.09) for the most extreme fire days.

## Discussion

We found consistent associations between fire-related PM_2.5_ and salbutamol dispensations. During the fire season a 10 ug/m^3^ increase in PM_2.5_ was associated with a 6% increase in salbutamol dispensations (RR = 1.06, 95% CI 1.04-1.07) in fire-affected populations, but no effect was observed in non-fire-affected populations. On extreme fire days the same PM_2.5_ increase was associated with a 7% increase in salbutamol dispensations in both types of populations (global RR = 1.07, 95% CI 1.04-1.09). To the best of our knowledge there is only one other study of pharmaceutical dispensations and forest fires, which evaluated the aftermath of an extreme fire season in Galacia, Spain. Caamano-Isorna et al. [16] reported that male and female pensioners (age not specified) increased consumption of medications to relieve obstructive lung disease by 10.3% and 12.1%, respectively, in the months following the severe fires, when compared with the previous months. No change was reported for non-pensioners.

Our results are consistent with other time-series studies on moderate to severe respiratory outcomes associated with exposure to forest fire smoke. Henderson et al. [9] reported that a 10 ug/m^3^ increase in PM_10_ was associated with a 6% increase in the odds of an asthma-specific physician visit (OR = 1.06, 95% CI 1.03-1.08) during the 2003 fire season in BC. In the state of Victoria, Australia, Tham et al. (2009) [32] reported that a 10 ug/m^3^ increase in PM_10_ was associated with a 2% increase in the relative rate (RR) of all respiratory ED visits (RR = 1.02, 95% CI 1.00-1.03). In Los Angeles, California, Delfino et al. [7] reported that a 10 ug/m^3^ increase in PM_2.5_ was associated with a 3% increase in the rate of all respiratory hospital admissions (RR = 1.03, 95% CI 1.01-1.04), a 5% increase in asthma admissions (RR = 1.05, 95% CI 1.02-1.08), and a 4% increase in chronic obstructive pulmonary disease admissions (RR = 1.04, 95% CI 1.00-1.08). Similarly, the Henderson et al. [9] study in BC also reported a 5% increase in the odds of all respiratory hospital admissions (OR = 1.05, 95% CI 1.00-1.10). Finally, in Sydney, Australia, Johnston et al. [33] reported that extreme smoke events were associated with a 9% increase in the odds of respiratory mortality, though the estimate was not statistically significant (OR = 1.09, 95% CI 0.88-1.36).

Because ambient PM_2.5_ monitors cannot differentiate between PM sources, we used empirical remote sensing data to objectively classify populations as fire-affected or non-fire-affected, and to identify extreme fire days. While several other studies have used satellite-based methods to identify smoke-affected periods and areas [1,7,9,34], our work is the first to leverage the fire radiative power measurements (proportional to smoke emissions) to classify populations and periods in this way. One might expect PM_2.5_ to have a clearer effect on salbutamol dispensations in LHAs where the smoke-related PM was extremely high, but this relationship was evident even in fire-affected LHAs with PM distributions that overlapped those of the non-fire-affected LHAs (Table 1). For example, the fire-affected LHA of Hope (population = 8,000; peak PM_2.5_ = 33.4 ug/m^3^; mean PM_2.5_ = 5.0 ug/m^3^) had a significant association (RR = 1.12, 95% CI 1.02-1.22), whereas the non-fire-affected LHA of Howe Sound (population = 32,000; peak PM_2.5_ = 33.7 ug/m3, mean PM_2.5_ = 8.2) did not (RR = 0.97, 95% CI 0.92-1.02). One outlier among the non-fire-affected LHAs was Kitimat (RR = 1.23, 95% CI 1.00-1.50), which is the site of an aluminum smelter.^a^ The population may therefore be exposed to PM_2.5_ that has a different toxicological profile than that in the other LHAs.

**Figure 5:**
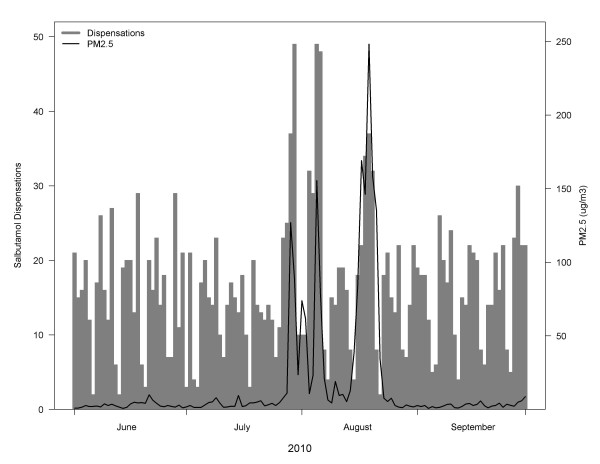
**Daily time-series of salbutamol dispensations compared with PM**_**2.5**_**concentrations in the Cariboo-Chilcotin LHA during the summer of 2010.** Days with low counts are weekends and holidays, when many pharmacies are closed.

Fire smoke often affects small populations because forest fires most commonly burn in rural and remote areas; extreme events that affect large cities are relatively rare. However, it has been challenging to find associations between more severe respiratory outcomes and smoke exposure in smaller populations. During the 2003 fire season in BC, Moore et al. [35] detected an increase in weekly respiratory physician visits in one larger community (approximate population 185,000) with heavy smoke, but not in a smaller nearby community (approximate population 110,000) with more moderate smoke. In Darwin, Australia (approximate population 110,000) Johnston et al. [10,33] conducted two studies of respiratory outcomes associated with ambient PM_10_ during the forest fire season. The first was an ecological study that detected significant increases in daily asthma ED visits only when concentrations were over 40 ug/m^3^[33]. The second was a case-crossover study on three years of hospital admissions [10]. Although the maximum daily concentration was 70 ug/m^3^, the positive associations were not statistically significant. In contrast, we have found strong and significant effects of PM_2.5_ on salbutamol dispensations in fire-affected populations ranging in size from 8,000 to 170,000 persons. Given that dispensations occur more frequently than severe outcomes, we suggest that they are more useful for studying the health effects of forest fire smoke in small populations. Furthermore, we observed that salbutamol dispensations rose rapidly in response to heavy smoke and fell rapidly as the smoke cleared (Figure 5), suggesting that dispensations may also be a responsive outcome for public health surveillance during smoke events.

There are important limitations to our analyses. First, this was an ecological study design, so we were unable to explore effect modification by individual factors. Second, a pharmaceutical dispensation does not necessarily reflect a disease exacerbation. Individuals with chronic lung disease may have sufficient reliever medication on hand, and not require a new dispensation for each exacerbation. Those who fill a prescription may do so for reasons related to the fire smoke (e.g., anticipated smoke effects), or for reasons that are completely unrelated (e.g., routine prescription renewal). Finally, we used data from single air quality monitoring stations to represent the exposure of populations within entire LHAs, some of which cover large geographic areas. Although most people in each LHA live in the monitored community, this homogenous approach to exposure assessment cannot account for the spatial variability inherent to fire smoke exposure.

Effective public health response to forest fire smoke events requires an understanding of its short-term health effects in order to identify who is most at risk, and to implement strategies to protect them. During milder events, the public health response may be limited to public education, but it should be rapidly escalated to provision of air shelters and/or evacuation as health risks increase. We have shown that pharmaceutical dispensations can be used to assess the population health effects in small communities. Given that these data are available in near-real-time, routine surveillance of pharmaceutical dispensations could play an important role in public health situational awareness and response. Further analyses are required to characterize short-term trends, and to create the indicators necessary to support fire smoke response guidelines.

## Conclusions

We report a clear association between fire-related PM_2.5_ and salbutamol dispensations in BC. The changes in salbutamol dispensations were observed in smaller populations than previously reported for any respiratory outcome (range: 8,000 to 170,000 persons, median: 26,000). This suggests that pharmaceutical dispensations can be leveraged in further research on acute respiratory events among small populations. Furthermore, this outcome was responsive to smoke-related PM_2.5_ concentrations, and may therefore be particularly useful for public health surveillance during forest fire smoke events.

## Endnotes

^a^ Note that the FRP time-series for Kitimat is the bottom panel of Figure 2, clearly showing little fire activity in the area.

## Abbreviations

PM_10_: PM less than 10 microns in diameter; PM_2.5_: PM less than 2.5 microns in diameter; RR: Rate ratio; CI: Confidence intervals; SABA: Short acting beta agonist; ED: Emergency department; BC: British Columbia; km^2^: Square kilometers; LHAs: Local health authority; FPR: Fire radiative power; MW: Megawatts; GIS: Geographic information system; FIRMS: Information Resource Management System; df: Degrees of freedom; *O*_*t*_: Observed dispensation count in the LHA on day *t*; *PM*_*lag01*_: PM_2.5_ concentration in the LHA averaged over days *t* (same day) and *t-1* (previous day); *T*_*t*_: Mean temperature in the region of the LHA on day *t*; *RH*_*t*_: Mean relative humidity in the region of the LHA on day *t*; *YMDOW*_*t*_: Year, month, and day of week (statutory holidays treated as Sundays) on day *t*; OR: Odds ratio.

## Competing interests

The authors declare that they have no competing interests.

## Authors’ contributions

CTE conceived of and helped design the study, critically reviewed the analysis and drafted the manuscript. SBH helped design the study, led the analysis, and helped draft the manuscript. VW assisted with data analysis and critically reviewed the manuscript. All authors read and approved the final manuscript.
